# Alzheimer’s Amyloid-β Accelerates Human Neuronal Cell Senescence Which Could Be Rescued by Sirtuin-1 and Aspirin

**DOI:** 10.3389/fncel.2022.906270

**Published:** 2022-06-17

**Authors:** Yi Li, Juan Lu, Yujun Hou, Shichao Huang, Gang Pei

**Affiliations:** ^1^School of Life Science and Technology, ShanghaiTech University, Shanghai, China; ^2^State Key Laboratory of Cell Biology, CAS Center for Excellence in Molecular Cell Science, Shanghai Institute of Biochemistry and Cell Biology, Chinese Academy of Sciences, University of Chinese Academy of Sciences, Shanghai, China; ^3^Institute for Regenerative Medicine, Shanghai East Hospital, Shanghai Key Laboratory of Signaling and Disease Research, School of Life Sciences and Technology, Tongji University, Shanghai, China; ^4^Shanghai Key Laboratory of Signaling and Disease Research, Collaborative Innovation Center for Brain Science, School of Life Sciences and Technology, Tongji University, Shanghai, China; ^5^Institute for Stem Cell and Regeneration, Chinese Academy of Sciences, Beijing, China

**Keywords:** Aβ, cell senescence, human neuronal cells, SIRT1, DNA damage

## Abstract

Cellular senescence is a major biological process related to aging. Neuronal cell senescence contributes to the pathogenesis of many aging-related neurodegenerative diseases including Alzheimer’s disease (AD). In this study, we showed that amyloid-β_42_ oligomers (Aβ), one of the core pathological players of AD, significantly upregulated the expression of senescence markers, p21, plasminogen activator inhibitor-1 (PAI-1), and SA-β-gal (senescence-associated β-galactosidase) in multiple human neuronal cells, including SK-N-SH cells, SH-SY5Y cells, and neural stem cell (NSC)-derived neuronal cells. Moreover, it was consistently observed among the cells that Aβ promoted senescence-associated DNA damage as the levels of 8-OHdG staining, histone variant H2AX phosphorylation (γ-H2AX), and genomic DNA lesion increased. Mechanism study revealed that the exposure of Aβ markedly suppressed the expression of sirtuin-1 (SIRT1), a critical regulator of aging, and the exogenous expression of SIRT1 alleviated Aβ-induced cell senescence phenotypes. To our surprise, a widely used cardiovascular drug aspirin considerably rescued Aβ-induced cellular senescence at least partially through its regulation of SIRT1. In conclusion, our findings clearly demonstrate that exposure of Aβ alone is sufficient to accelerate the senescence of human neuronal cells through the downregulation of SIRT1.

## Introduction

Alzheimer’s disease (AD) is an age-associated, progressive, and irreversible neurodegenerative disorder that exponentially increased with age (Baker and Petersen, 2018; Liu, 2022). Cell senescence, with the classical phenotypic hallmarks include senescence-associated β-galactosidase (SA-β-gal), cell cycle arrest, persistent DNA damage response (DDR), and senescence-associated secretory phenotype (SASP) including inflammatory cytokines, growth factor, matrix metalloproteinases, and other proteinases (Coppé et al., 2010; Cuollo et al., 2020; Fafián-Labora and O’Loghlen, 2020), has been demonstrated to play an important role in onset and aggravation of AD (Martínez-Cué and Rueda, 2020). Recent studies have shown that plasminogen activator inhibitor-1 (PAI-1) also presents a key marker of cell senescence and contributes to various aging-associated morbidities (Gerenu et al., 2017; Vaughan et al., 2017; Kritsilis et al., 2018; Bryant et al., 2020; Tang et al., 2022). Mounting evidence has demonstrated that disease-associated microglia display several features of senescence and preventing microglia from senescence could lead to reduced amyloidosis and synaptic damage in age-related AD (Daria et al., 2017; Hu et al., 2021; Hu et al., 2022). Besides, it has been found that Aβ oligomers, one of the major players in AD, induced senescence in oligodendrocyte progenitor cells and in adult hippocampal neural stem/progenitor cells (He et al., 2013; Zhang et al., 2019). More importantly, the clearance of senescent cells was reported to decrease Aβ plaque size and improve cognition, further demonstrating senescent cells accelerate Aβ pathology (Zhang et al., 2019). However, neuronal senescence in AD models is relatively complicated, and there is a growing concern about cell senescence in brain post-mitotic cells. Many evidence demonstrated that terminally differentiated neurons show some phenotypes similar to senescence, such as cell cycle arrest, SA-β-gal activity, lipofuscin, DNA damage response, and activation of SASP both *in vitro* and *in vivo* (Wong et al., 2009; Geng et al., 2010; Jurk et al., 2012; Ota et al., 2012; Kang et al., 2015; Forero et al., 2016; Rocchi et al., 2021). Therefore, preventing neuronal cells from senescence might be beneficial in the prevention and treatment of age-related neurodegenerative disorders, such as AD.

Sirtuins family, as NAD^+^-dependent deacetylases, plays the important roles in delaying cellular aging and extending the life cycle of organs through various complex cellular signaling regulation (Satoh et al., 2011; Zhao et al., 2020). Among the mammalian sirtuins, SIRT1 is widely recognized as a regulator of cellular and organismal processes, including gene regulation, genome stability maintenance, metabolism, autophagy, senescence, and tumorigenesis (Oberdoerffer et al., 2008; Zhang and Kraus, 2010; Xu J. et al., 2018; Xu et al., 2020). An increasing number of evidence suggests that elevated SIRT1 has beneficial effects on aging-related diseases. SIRT1 is involved in protecting endothelial cells from stress-induced premature senescence and replicative senescence (Ota et al., 2007; Zu et al., 2010; Wan et al., 2014). Also, knockdown of SIRT1 induced cell senescence and inhibited cell proliferation in young mesenchymal stem cells (hMSCs) while overexpression of SIRT1 resulted in delayed senescence in primary human lung fibroblasts and aged MSCs (Huang et al., 2008; Chen et al., 2014; Pi et al., 2021). However, whether SIRT1 can regulate human neuronal senescence and counteract Aβ-induced neuronal senescence is not well established. Aspirin, a widely used cardiovascular drug, was shown to reduce endothelial cell senescence and ameliorate doxorubicin-induced cell senescence in human and mouse fibroblasts (Bode-Böger et al., 2005; Feng et al., 2019), whereas the effect of aspirin on neuronal cell senescence is unknown in AD. In this study, we found that Aβ accelerated cell senescence in SK-N-SH cells, SH-SY5Y cells, and NSC-derived neuronal cells. Stimulation of neuronal cells with Aβ induced the reduction of SIRT1, and the exogeneous expression of SIRT1 could significantly alleviate Aβ-induced cell senescence, indicating Aβ-induced neuronal senescence through downregulation of SIRT1. To our surprise, we found aspirin could partially prevent human neuronal cell senescence by upregulating SIRT1.

## Materials and Methods

### Cell Culture

SK-N-SH cells and SH-SY5Y cells were purchased from ATCC. SK-N-SH cell line was cultured in modified Eagle’s medium (MEM), and SH-SY5Y cell line was cultured in MEM/F12 Medium, with 10% fetal bovine serum (FBS) and 100 U/ml penicillin and 0.1 mg/ml streptomycin in a humidified incubator with 5% CO2/95% air (v/v) at 37°C.

### Human Neural Stem Cell Differentiation

Human induced pluripotent stem cell (iPSC)-derived NSCs (IxCell Biotechnology, Ltd.) were maintained as adherent culture in the medium, containing 50% Dulbecco’s modified Eagle medium: nutrient mixture F-12 (DMEM/F12, Gibco), 50% neurobasal (Gibco), 1*N2 supplement (Gibco), 1*B27 supplement (Gibco), 1*MEM non-essential amino acids solution, 1*GlutaMAX (Gibco), 10 ng/μl bFGF, 10 ng/μl hlif, 3 μM CHIR99021 (Selleckchem), 5 μM SB431542 (Selleckchem), and 200 μM ascorbic acid (Sigma) and cultured in a humidified incubator with 5% CO2/95% air (v/v) at 37°C. For differentiation, the human NSCs were seeded in 24-well plates coated by poly-D-lysine and laminin, at the density of 5*10^4^ cells per well. On the second day, medium was changed to neuron differentiation medium, neurobasal with 1*B27, 1*CultureOne supplement (ThermoFisher Scientific), 1*GlutaMAX, and 200 μM ascorbic acid. The differential medium was refreshed every other day (Lu et al., 2019). At the day 12 of differentiation, cells were treated by Aβ for 48 h.

### Plasmid Construction, Transfection, and siRNA

SIRT1 cDNA was generated from pCMV-SIRT1-t1-Flag (purchased from Sino Biological) *via* PCR amplification and then cloned into the FuGW vector by using Seamless Cloning Kit (Beyotime, D7010M) according to the manufacturer’s instructions and confirmed by DNA sequencing. Cells were transfected with constructed SIRT1 plasmid using lipofectamine 3000 (ThermoFisher Scientific, L3000001). After transfection for 24 h, cells were stimulated by Aβ. The knockdown of SIRT1 was performed by the transfection with specific siRNA (Tsingke Biotechnology Co., Ltd.) using lipofectamine 3000.

The cloning primers were as follows:

Forward, TGGGCTGCAGGTCGACTCTAGAATGGCA GATGAAGCAGCTCTC;

Reverse, TTGATATCGAATTCTAGACTATGATTTGTTTGA TGGATAGTTCATGTCT;

The siRNA primers were as follows:

siSIRT1-1: Forward, CACCUGAGUUGGAUGAUAUTT;

siSIRT1-1: Reverse, AUAUCAUCCAACUCAGGUGTT;

siSIRT1-2: Forward, GUCUGUUUCAUGUGGAAUATT;

siSIRT1-2: Reverse, UAUUCCACAUGAAACAGACTT;

### Aβ_42_ Oligomer Preparation

Aβ_42_ oligomers (Aβ) were prepared according to the previous publications (Li et al., 2020). In brief, 2 mg Aβ_42_ peptides (CHINESE PEPTIDE, AMYD-003) dissolved in 2 ml cold hexafluoroisopropanol (HFIP) (Sigma) were dispensed into Protein LoBind tubes (Eppendorf, 030108094) and dried overnight at room temperature (RT). HFIP-treated Aβ_42_ peptides were resuspended in dimethyl sulfoxide (DMSO) and diluted in phenol-red free DMEM/F12 medium to obtain a 100 μM stock solution. The diluted Aβ_42_ peptides were vortexed for 15 s followed by incubation for 24 h at 4°C.

### Cell Growth

SK-N-SH or SH-SY5Y cells were seeded in a 96-well plate at 1 * 10^4^ cells/well. The cells were treated with Aβ or aspirin at the indicated time. After the treatment for 72 h, cell viability was detected using luminescent cell viability assay (Vazyme, DD1101-02), following the manufacturer’s guideline, and then were measured by BioTek SynergyNEO (BioTek, United States).

### Long-Range PCR Lesion Assay

Genomic DNA was extracted from SK-N-SH or SH-SY5Y cells treated by Aβ for 72 h using TIANamp Genomic DNA kit (Tiangen, DP304-03), according to the manufacturer’s instruction. The samples were quantified with Nanodrop (Gene Company Limited) and then diluted to 5 ng/μl. About 20 ng DNA as the template was used for quantitative PCR-based amplification nuclear DNA segment using TaKaRa LA Taq DNA Polymerase (TaKaRa LA). A small segment, 175-bp was amplified using 2*Taq PCR Master Mix (Tiangen) as an inner control.

The primers used are as follows:

12.2 kb Forward, CATGTCACCACTGGACTCTGCAC;

12.2 kb Reverse, CCTGGAGTAGGAACAAAAATTGCTG;

13.5 kb Forward, CGAGTAAGAGACCATTGTGGCAG;

13.5 kb Reverse, GCACTGGCTTTAGGAGTTGGACT;

175 bp Forward, GGGATAACATCCAGGGCATT;

175 bp Reverse, CCCTGACGTTTTAGGGCATA;

### Senescence-Associated -β-Galactosidase (SA-β-Gal) Staining Assay

SA-β-gal staining of SK-N-SH or SH-SY5Y cells was performed as previously reported (Li et al., 2020). Cells were treated by Aβ or pre-incubated aspirin for 6 h with EX727 or without, followed by Aβ challenge for 72 h. Briefly, after treatment, cells were washed two times with phosphate buffer solution (PBS), and then stained using Senescence β-Galactosidase Staining Kit for 12 h according to the manufacturer’s guideline (Beyotime, C0602). Images were captured with Olympus IX73 microscope. The numbers of SA-β-gal-positive cells were measured with Fiji.

### Reverse Transfection and Quantitative Real-Time PCR

After treatment with Aβ at the indicated time, cells were harvested and total RNAs were isolated using an EZ-press RNA purification kit (EZBioscience, B0004D) according to the manufacturer’s instructions. Reverse transfection was performed using PrimeScript RT Master Mix (Takara, RR036) under the following conditions: 37°C, 15 min and 85°C, 15 s. Then, gene transcripts were analyzed by quantitative real-time PCR conducted with 2x HotStart SYBR Green qPCR Master Mix (ExCell Bio, MB000-3013) on a Stratagene Mx3000P (Agilent Technologies). The reaction parameters were as follows: 95°C for 10 min; 95°C for 30 s, 40 cycles; 60°C for 30 s; and 72°C for 30 s. An additional cycle was performed for evaluation of primer’s dissociation curve: 95°C for 1 min, 60°C for 30 s, and 95°C for 30 s. Each cDNA sample was amplified two times.

The primer sequences used were as follows:

p21-Forward, CGATGGAACTTCGACTTTGTCA;

p21-Reverse, GCACAAGGGTACAAGACAGTG;

PAI-1-Forward, ACCGCAACGTGGTTTTCTCA;

PAI-1-Reverse, TTGAATCCCATAGCTGCTTGAAT;

MMP3-Forward, CTGCTGTTGAGAAAGCTCTG;

MMP3-Reverse, AATTGGTCCCTGTTGTATCCT;

p53-Forward, CCCCTCCTGGCCCCTGTCATCTTC;

p53-Reverse, GCAGCGCCTCACAACCTCCGTCAT;

Δ133p53-Forward, TGACTTTCAACTCTGTCTCCTTCCT;

Δ133p53- Reverse, GGCCAGACCATCGCTATCTG;

IL6-Forward, GTGGCTGCAGGACATGACAA;

IL6-Reverse, TGAGGTGCCCATGCTACATTT;

HPRT-Forward, CCTGGCGTCGTGATTAGTGAT;

HPRT-Reverse, AGACGTTCAGTCCTGTCCATAA;

### Immunofluorescence Staining

Immunostaining was performed as previously described with minor modification (Gao et al., 2019). Cells were treated by Aβ or pre-incubated aspirin for 6 h followed by Aβ challenge for 72 h. Briefly, cells for immunostaining were washed two times with PBS and fixed with 4% PFA for 15 min at RT. Then, cells were treated with permeabilization and blocking buffer (1% BSA and 0.3% Triton-X 100 in PBS) for 1 h at RT. Primary antibodies diluted in buffer (1% BSA and 0.3% Triton-X 100 in PBS) were incubated overnight at 4°C. Cells were washed at least three times with PBS and incubated with fluorescent-dye conjugated secondary antibodies at RT for 1h, followed by incubation with DAPI (1:3000, Beyotime) for 15 min. Slides were mounted and images were captured with Leica TCS SP8 WLL microscope (Leica, German). Antibodies used for immunofluorescence analysis are as follows: anti-γ-H2AX (1:800, CST), anti-8-OHdG (1:1000, Rockland), anti-Tuj-1 (1:500, Abcam), and anti-Sox2 (1:200, R&D, system).

### Western Blot

Western blot was performed following previous publications with minor modification. After cells were incubated with indicated treatment, total cell lysates were separated by 12% sodium dodecyl sulfate-polyacrylamide gel electrophoresis (SDS-PAGE) and transferred onto nitrocellulose membranes (400 mA constant current, 2 h, 4°C). Membranes were blocked with 5% non-fat milk in TBS containing 0.1% Tween-20 for 45 min at RT. Subsequently, membranes were treated with antibodies. Antibodies were used as follows: anti-p21 (1:1000, CST, 2947S), anti-PAI-1 (1:1000, CST, 11907), anti-p53 (1:1000, CST, 2527), anti-γ-H2AX (1:1000, CST, 9718), anti-SIRT1 (1:1000, CST, 8469), anti-SIRT5 (1:1000, Proteintech, 15122-1-AP), and actin (1:1000, Sigma, A2066).

### Statistical Analysis

All data were analyzed using Prism 8.0. All quantified data were presented as mean ± SEM. Unpaired Student’s *t*-test (two-tailed) was applied for the comparisons of two datasets and one-way or two-way analysis of variance (ANOVA) with Bonferroni’s *post*-test was used where more than two datasets or groups were compared. Statistical significance was accepted at *p* < 0.05.

## Results

### Aβ Accelerated Cell Senescence in Human Neuronal Cells

**FIGURE 1:**
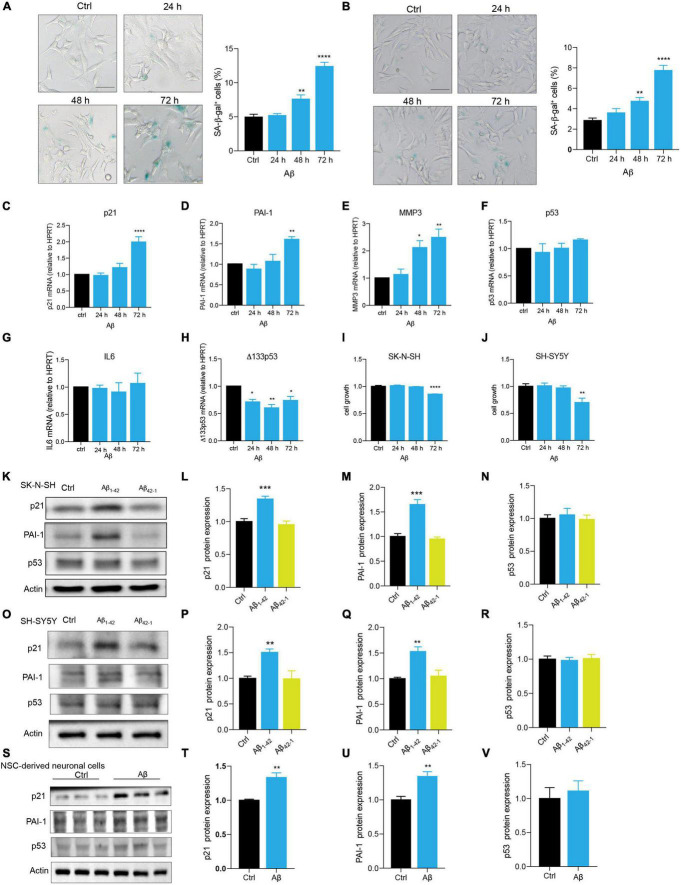
Aβ accelerated cell senescence in human neuronal cells. **(A)** The representative images of SA-β-gal staining in SK-N-SH cells treated by Aβ (5 μM) at indicated time, and quantification of relative number of SA-β-gal-positive cells. The images were captured by Olympus IX73. Scale bars, 50 μm. **(B)** The representative images of SA-β-gal staining in SH-SY5Y cells challenged by Aβ (5 μM) at indicated time, and quantification of relative number of SA-β-gal-positive cells. The images were captured by Olympus IX73. Scale bars, 50 μm. **(C-H)** p21 **(C)**, PAI-1 **(D)**, MMP3 **(E)**, p53 **(F)**, IL6 **(G)**, Δ133p53 **(H)** mRNA levels were measured after treatment with Aβ (5 μM) at indicated time. **(I-J)** Cell growth was detected by luminescent cell viability assay in SK-N-SH cells **(I)** and SH-SY5Y cells **(J)** challenged by Aβ (5 μm) at indicated time. **(K-N)** Cells were incubated with Aβ_1–42_ (5 μM) or Aβ_42–1_ (5 μM) for 72 h in SK-N-SH cells, and cell lysates were prepared and analyzed using western blotting with p21, PAI-1, p53 antibody. Actin was used as a loading control. Quantification of relative p21 **(L)**, PAI-1 **(M)**, p53 **(N)** protein level in **(K)**. **(O-R)** Cells were incubated with Aβ_1–42_ (5 μM) or Aβ_42–1_ (5 μM) for 72 h in SH-SY5Y cells, and cell lysates were prepared and analyzed using western blotting with p21, PAI-1, and p53 antibody. Actin was used as a loading control. Quantification of relative p21 **(P)**, PAI-1 **(Q)**, p53 **(R)** protein level in **(O)**. **(S-V)** Cells were incubated with Aβ (5 μM) for 48 h in NSC-derived neuronal cells, and cell lysates were prepared and analyzed using western blotting with p21, PAI-1, and p53 antibody. Actin was used as a loading control. Quantification of relative p21 **(T)**, PAI-1 **(U)**, p53 **(V)** protein level in **(S)**. The data are presented as mean ± SEM, *n* ≥ 3 independent experiments, *p < 0.05, **p < 0.01, ***p < 0.001, and ****p < 0.0001, analyzed by one-way ANOVA followed by Bonferroni test or unpaired Student’s *t*-test (two-tailed).

Neuronal cell senescence was examined by the detection of SA-β-gal activity, cell cycle arrest characterized by upregulation of p16, p21, and p53, PAI-1 expression, and SASP-related MMP3 and IL6. Compared with control, Aβ treatment significantly increased SA-β-gal-positive cells in dose- and time-dependent manners (Supplementary Figures 1A,B and Figure 1A) in SK-N-SH cells. This time dependence was consistently observed in another human neuronal cell line, SH-SY5Y cells (Figure 1B). Here, treatment with Aβ obviously upregulated the mRNA levels of cell senescence markers, p21 (Figure 1C), PAI-1 (Figure 1D), and MMP3 (Figure 1E), but not IL6 (Figure 1G). Although there was no change in p53 mRNA level (Figure 1F), Δ133p53, the isoform of p53, was markedly declined (Figure 1H). To further confirm, we detected the protein expression of the main cell senescence markers, p21, PAI-1, and p53 after the treatment with Aβ for 72 h. Consistent with mRNA results, Aβ significantly induced p21 and PAI-1 protein expression, but not p53 in SK-N-SH cells (Figures 1K-N) and SH-SY5Y cells (Figures 1O-R). By contrast, Aβ_42–1_, as a negative control, had no obvious effect (Figures 1K-R). To examine this in a more relevant cell model, we differentiated NSC to neuronal cells (Supplementary Figure 1C), and as expected, Aβ increased the expression of cell senescence genes in SK-N-SH cells, SH-SY5Y cells, and NSC-derived neuronal cells (Figures 1S-V). The effect of Aβ treatment on cell growth was also detected and data showed that Aβ significantly decreased cell number at 72h in SK-N-SH cells (Figure 1I) and SH-SY5Y cells (Figure 1J). These findings revealed that Aβ is sufficient to accelerate human neuronal cell senescence.

### Aβ Induced Senescence-Associated DNA Damage in Human Neuronal Cells

**FIGURE 2:**
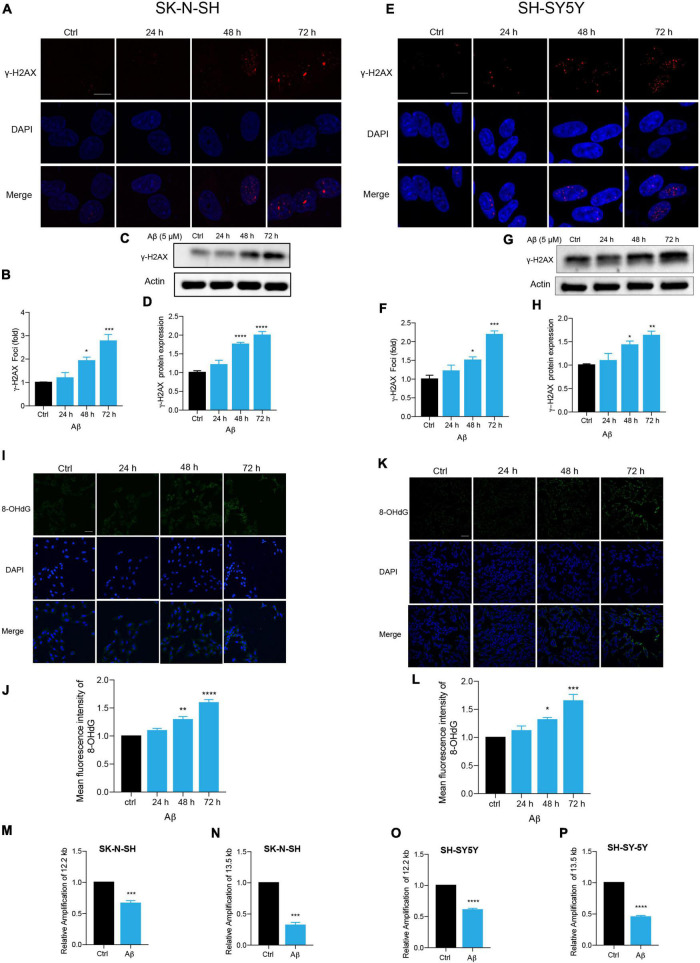
Aβ induced senescence-associated DNA damage in human neuronal cells. **(A,B)** Representative images of DAPI and γ-H2AX fluorescent staining in Aβ (5 μM)-treated SK-N-SH cells at 24, 48, and 72 h. The quantificative analysis of γ-H2AX foci number in **(B)**. The pictures were captured by Leica TCS SP8 WLL. Scale bar, 10 μm. **(C,D)** Cells were incubated with Aβ for indicated time in SK-N-SH cells and western blot analysis of γ-H2AX protein in cell lysates **(C)**. Actin was used as a loading control. Quantification of γ-H2AX protein level in SK-N-SH cells **(D)**. **(E,F)** Representative images of DAPI and γ-H2AX fluorescent staining in Aβ-treated SH-SY5Y cells at 24, 48, and 72 h. The quantificative analysis of γ-H2AX foci number in SK-N-SH cells **(F)**. The pictures were captured by Leica TCS SP8 WLL. Scale bar, 10 μm. **(G,H)** Cells were incubated with Aβ for indicated time in SH-SY5Y cells, and western blot analysis of γ-H2AX protein in cell lysates **(G)**. Actin was used as a loading control. Quantification of γ-H2AX protein level **(H)**. **(I,J)** Representative images of 8-OHdG staining in SK-N-SH cells treated by Aβ (5 μM) at indicated time **(I)**. The quantification of 8-OHdG fluorescent intensity **(J)**. The pictures were obtained by Leica TCS SP8 WLL. Scale bar, 50 μm. **(K,L)** Representative images of 8-OHdG staining in SH-SY5Y cells treated by Aβ (5 μM) at indicated time **(K)**. The quantification of 8-OHdG fluorescent intensity **(L)**. The pictures were obtained by Leica TCS SP8 WLL. Scale bar, 50 μm. **(M,N)** Long-range PCR base-assessment of nDNA (12.2 kb) **(M)** and nDNA (13.5 kb) **(N)** damage in SK-N-SH cells incubated with Aβ (5 μM) for 72 h. 175 bp as inner control. **(O,P)** Long-range PCR base assessment of nDNA (12.2 kb) **(O)** and nDNA (13.5 kb) **(P)** damage in SH-SY5Y cells incubated with Aβ (5 μM) for 72 h. 175 bp as an inner control. The data are presented as mean ± SEM, *n* ≥ 3 independent experiments, *p < 0.05, **p < 0.01, ***p < 0.001, and ****p < 0.0001, analyzed by one-way ANOVA followed by Bonferroni test or unpaired Student’s *t*-test (two-tailed).

Persistent DDR is a characteristic feature of senescent cells (von Zglinicki et al., 2005; Chen et al., 2007; d’Adda di Fagagna, 2008; Kumari and Jat, 2021). DNA double-strand break (DSB) is the most dangerous type of DNA damage, and phosphorylation of H2AX, called γ-H2AX, in the position of Ser139 occurs in response to DSB formation (Mah et al., 2010; Podhorecka et al., 2010). Here, immunostaining of γ-H2AX presented increased DNA damage foci numbers by Aβ treatment in SK-N-SH cells (Figures 2A,B). Meanwhile, an increased protein expression of γ-H2AX was detected after Aβ treatment (Figures 2C,D). The location and expression of γ-H2AX were further verified in SH-SY5Y by immunostaining (Figures 2E,F) and western blot analysis (Figures 2G,H) and in NSC-derived neuronal cells by western blot (Supplementary Figures 2A,B). Oxidative DNA damage was evaluated by immunostaining of 8-hydroxy-2’deoxyguanosine (8-OHdG), a major oxidative DNA adduct involved in senescence and many disease processes (Nakae et al., 2000). The results revealed that Aβ markedly increased fluorescence intensity of 8-OHdG in SK-N-SH (Figures 2I,J) and SH-SY5Y cells (Figures 2K,L). Genomic DNA damage was then evaluated by long-range PCR lesion assay. A large nuclear segment is amplified with an efficiency that can be declined by the lesions of the DNA template. The results showed that Aβ significantly reduced the relative amplification of nuclear (12.2 and 13.5 kb) genomic DNA in SK-N-SH (Figures 2M,N) and SH-SY5Y cells (Figures 2O,P). The results were further confirmed by agarose gel electrophoresis (Supplementary Figure 2C). To sum up, these results suggested that Aβ accelerated DNA damage in human neuronal cells, which might contribute to cell senescence.

### Aβ Induced Neuronal Cell Senescence Through Suppressing SIRT1 Expression

**FIGURE 3:**
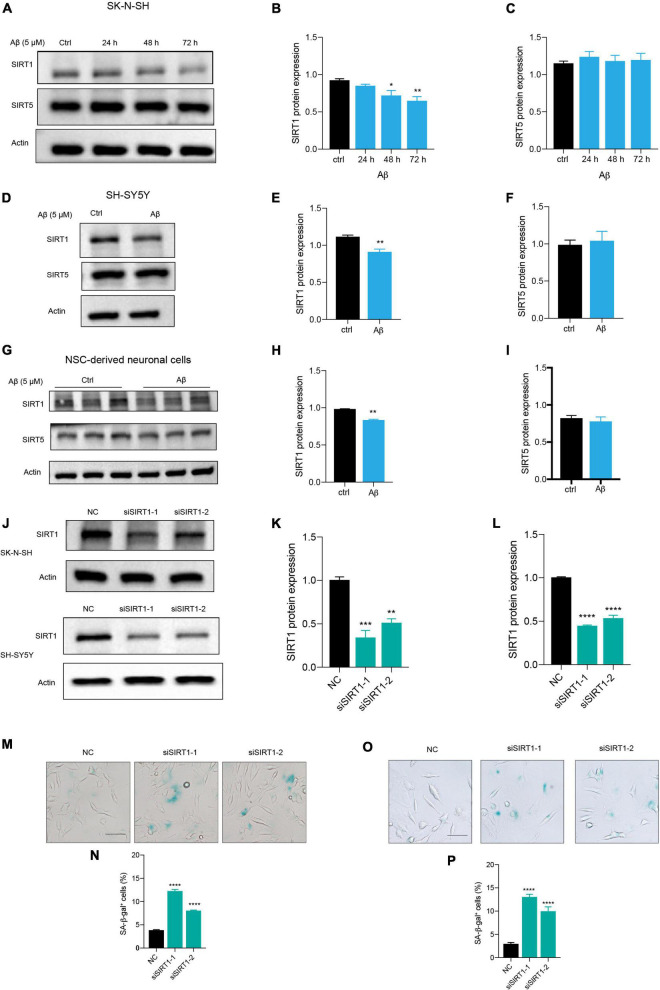
Aβ induced neuronal cell senescence through suppressing SIRT1 expression. **(A)** Cells were incubated with Aβ for 24, 48, and 72 h in SK-N-SH cells, and cell lysates were prepared and analyzed using western blotting with SIRT1 and SIRT5 antibody. Actin was used as a loading control. **(B,C)** Quantification SIRT1 **(B)** and SIRT5 **(C)** protein level in **(A)**. **(D)** Cells were challenged with Aβ for 72 h in SH-SY5Y cells, western blot analysis of SIRT1 and SIRT5 protein. Actin was used as a loading control. **(E,F)** Quantification SIRT1 **(E)** and SIRT5 **(F)** protein level in **(D)**. **(G)** Cells were treated with Aβ for 48 h in NSC-derived neuronal cells and western blot analysis of SIRT1 and SIRT5. Actin was used as a loading control. **(H,I)** Quantification SIRT1 **(H)** and SIRT5 **(I)** protein level in **(G)**. **(J)** Cells were transfected with negative control (NC), siSIRT1-1, siSIRT1-2 in SK-N-SH and SH-SY5Y cells, and harvested after 72 h. Western blot analysis of SIRT1 protein level. Actin was used as a loading control. **(K,L)** Quantification of SIRT1 protein level in SK-N-SH cells **(K)** and in SH-SY5Y cells **(L)**. **(M,N)** The representative images of SA-β-gal staining in SK-N-SH cells transfected with siSIRT1 and stained after 72 h in **(M)**. The quantification analysis of SA-β-gal-positive cells in **(N)**. **(O,P)** The representative images of SA-β-gal staining in SH-SY5Y cells transfected with siSIRT1 and treated for 72 h in **(O)**. The quantification analysis of SA-β-gal-positive cells in **(P)**. The data are presented as mean ± SEM, *n* ≥ 3 independent experiments, *p < 0.05, **p < 0.01, ***p < 0.001, and ****p < 0.0001, analyzed by one-way ANOVA followed by Bonferroni test or unpaired Student’s *t*-test (two-tailed).

Among the sirtuins, we found that Aβ declined SIRT1, SIRT3, and SIRT6 protein levels in human neuronal cells, but the downregulation of SIRT1 is mostly remarkable (data not shown). The protein level of SIRT1 was significantly reduced after Aβ stimulation in a time-dependent manner, compared to the control in SK-N-SH cells (Figures 3A,B). By contrast, the SIRT5 expression was not changed (Figures 3A,C). The same results were obtained in SH-SY5Y cells (Figures 3D,E,F) and NSC-derived neuronal cells (Figures 3G,H,I). To further elucidate whether downregulation of SIRT1 plays a role in human neuronal cell senescence, we tested whether loss of SIRT1 expression increased senescence process of neuronal cells by the application of siRNA that specifically targets its transcript. Transfection of SK-N-SH cells with siSIRT1-1 or siSIRT1-2 successfully downregulated protein level of SIRT1 assessed by western blot (Figures 3J,K). This was consistently observed in SH-SY5Y cells (Figures 3J,L). The knockdown of SIRT1 resulted in a remarkable increase in the percentage of SA-β-gal-positive cells in SK-N-SH (Figures 3M,N) and SH-SY5Y cells (Figures 3O,P). These findings suggested that SIRT1 may be involved in human neuronal senescence and Aβ accelerated neuronal senescence by modulating SIRT1.

### Exogenous Expression of SIRT1 Rescued Aβ-Induced Cell Senescence

**FIGURE 4:**
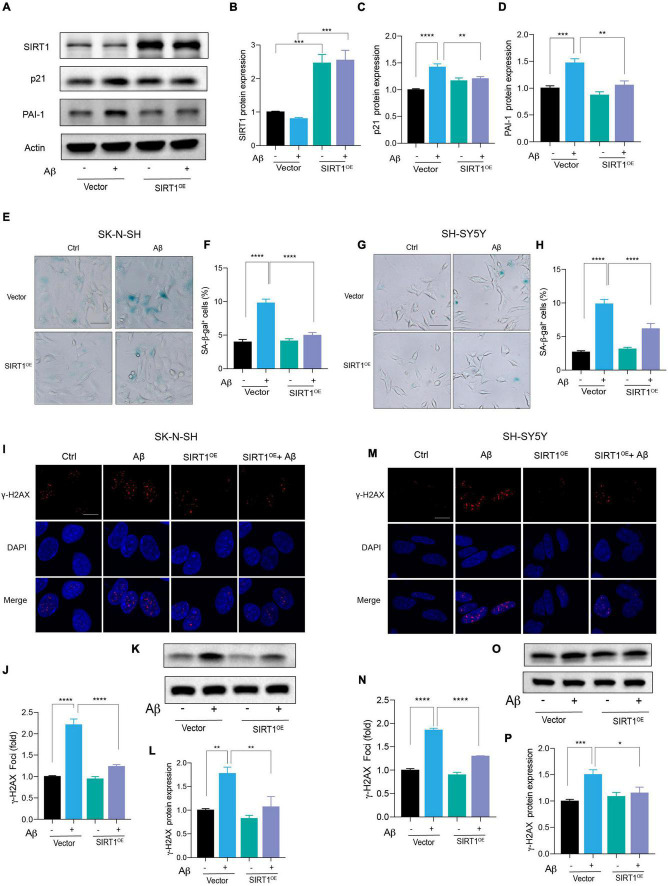
Exogenous expression of SIRT1 rescued Aβ-induced cell senescence. **(A)** Cells were transfected with Vector and SIRT1 plasmid in SK-N-SH cells, and after 24 h transfection, cells were incubated with Aβ or without for another 72 h. Western blot analysis of SIRT1, p21, PAI-1 protein level. Actin was used a loading control. **(B-D)** Quantification of SIRT1 **(B)**, p21 **(C)**, and PAI-1 **(D)** protein level in (A). **(E,F)** The representative images of SA-β-gal staining in SK-N-SH cells treated with Aβ or without for 72 h, after transfected with Vector and SIRT1 plasmid for 24 h **(E)**. Quantification of relative number of SA-β-gal-positive cells in **(F)**. The pictures were obtained by Olympus IX73. Scale bar, 50 μm. **(G,H)** The representative images of SA-β-gal staining in SH-SY5Y cells incubated with Aβ or without for 72 h, after transfected with Vector and SIRT1 for 24 h **(G)**. Quantification of relative number of SA-β-gal-positive cells in **(H)**. The pictures were obtained by Olympus IX73. Scale bar, 50 μm. **(I-J)** Representative images of DAPI and γ-H2AX fluorescent staining in SK-N-SH cells transfected with SIRT1 plasmid for 24 h and then incubated with Aβ or without for another 72 h in **(I)**. The quantification of relative γ-H2AX foci number in SK-N-SH cells in **(J)**. **(K,L)** Western blot analysis of γ-H2AX protein in SK-N-SH cells transfected with SIRT1 plasmid for 24 h and then incubated with Aβ or without for another 72 h **(K)**. The quantification of γ-H2AX protein level in **(L)**. **(M,N)** Representative images of DAPI and γ-H2AX in SH-SY5Y cells transfected with SIRT1 plasmid for 24 h and followed with Aβ or without for another 72 h in **(M)**. The quantification of relative γ-H2AX foci number in **(N)**. **(O,P)** Western blot analysis of γ-H2AX protein in SH-SY5Y cells transfected with SIRT1 plasmid for 24 h and followed with Aβ or without for another 72 h **(O)**. The quantification of γ-H2AX protein level in **(P)**. The data are presented as mean ± SEM, n ≥ 3 independent experiments, *p < 0.05, **p < 0.01, ***p < 0.001, and ****p < 0.0001, analyzed by one-way ANOVA followed by Bonferroni test.

Based on the above results, we suspected that the introduction of SIRT1 in human neuronal cells could alleviate Aβ-induced cell senescence. Therefore, the SIRT1 plasmid was constructed and transfected into neuronal cells. The successful overexpression of SIRT1 protein was confirmed by western blot (Figures 4A,B). The upregulation of SIRT1 significantly decreased p21 protein level under stimulation with Aβ (Figures 4A,C). Particularly, exogeneous SIRT1 almost rescued the Aβ-reduced PAI-1 protein levels to the untreated control (Figures 4A,D). Additionally, the number of SA-β-gal-positive cells in Aβ-treated neuronal cells was remarkably declined by SIRT1 overexpression. The results were verified in both SK-N-SH (Figures 4E,F) and SH-SY5Y (Figures 4G,H) cells. Finally, we assessed the effect of SIRT1 overexpression on Aβ-induced DNA damage response. The number of γ-H2AX foci was obviously decreased detected by immunofluorescence, and the protein level of γ-H2AX was also declined examined by western blot both in SK-N-SH (Figures 4I-L) and SH-SY5Y cells (Figures 4M-P). These results revealed that overexpression of SIRT1 can alleviate Aβ-induced cell senescence and senescence-associated DNA damage in human neuronal cells.

### Aspirin Upregulated SIRT1 to Alleviate Aβ-Induced Senescence

**FIGURE 5:**
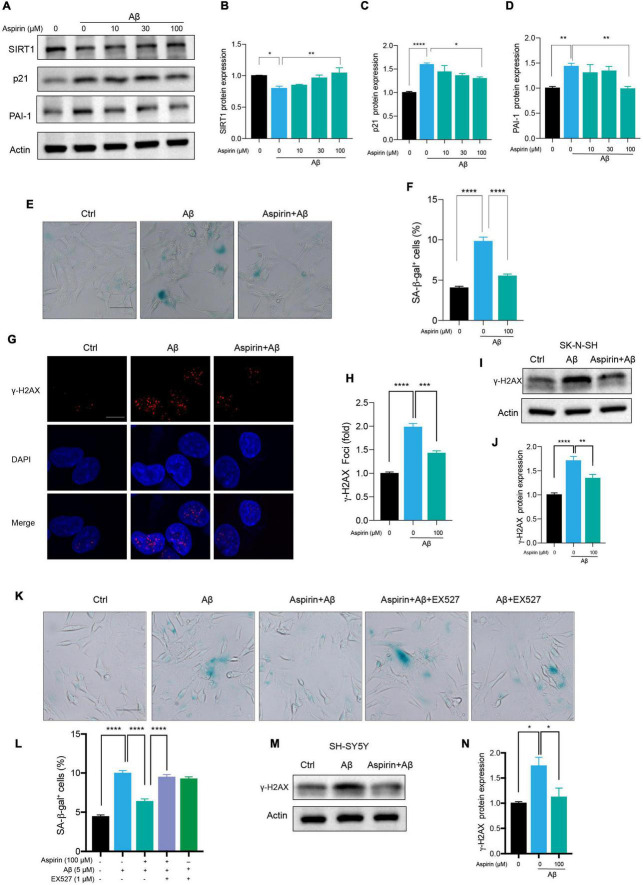
Aspirin upregulated SIRT1 to alleviate Aβ-induced senescence. **(A)** Cells were incubated with aspirin at indicated dose for 6 h and then challenged with Aβ (5 μM) for another 72 h. Western blot analysis of SIRT1, p21, and PAI-1 protein level. Actin was used a loading control. **(B-D)** Quantification of SIRT1 **(B)**, p21 **(C)**, PAI-1 **(D)** protein level in **(A)**. **(E,F)** The representative images of SA-β-gal staining in SK-N-SH cells pre-incubated with aspirin (100 μM) for 6 h followed by Aβ (5 μM) challenge for 72 h **(E)**. The quantification of relative number of SA-β-gal-positive cells in **(F)**. The pictures were captured by Olympus IX73. Scale bars, 50 μm. **(G,H)** Representative images of γ-H2AX fluorescent staining in SK-N-SH cells pre-incubated with aspirin (100 μM) for 6 h followed by Aβ (5 μM) challenge for 72 h **(G)**. The quantification of relative γ-H2AX foci number in **(H)**. **(I,J)** Western blot analysis of γ-H2AX protein level in SK-N-SH cells pre-incubated with aspirin (100 μM) for 6 h followed by Aβ (5 μM) challenge for 72 h **(I)**. The quantification of γ-H2AX protein level in **(J)**. **(K,L)** The representative images of SA-β-gal staining in SK-N-SH cells pre-incubated with aspirin (100 μM) for 6 h with EX527 (1 μM) (SIRT1 inhibitor) or without, followed by Aβ (5 μM) challenge for 72 h **(K)**. The quantification of relative number of SA-β-gal-positive cells in **(L)**. The pictures were captured by Olympus IX73. Scale bars, 50 μm. **(M,N)** Western blot analysis of γ-H2AX protein level in SH-SY5Y cells pre-incubated with aspirin (100 μM) for 6 h followed by Aβ (5 μM) challenge for 72 h **(M)**. The quantification of γ-H2AX protein level in **(N)**. The data are presented as mean ± SEM, *n* ≥ 3 independent experiments, *p < 0.05, **p < 0.01, ***p < 0.001, and ****p < 0.0001, analyzed by one-way ANOVA followed by Bonferroni test.

Small-scale screening was used to find compounds that can slow cell senescence in human neuronal cells. To our surprise, aspirin, a widely used cardiovascular drug, inhibited Aβ-induced cell senescence. First, we found that aspirin which had no effect on cell viability at indicated dosage (Supplementary Figure 2D), dose-dependently declined the protein levels of p21 and PAI-1 in Aβ-treated neuronal cells (Figures 5A,C,D). Aspirin also increased SIRT1 protein expression in a dosage-dependent manner (Figures 5A,B). Next, we observed that the number of SA-β-gal-positive cells in Aβ-treated neuronal cells was remarkably declined by the pre-treatment with aspirin (Figures 5E,F). In addition, aspirin significantly decreased the proportion of γ-H2AX foci (Figures 5G,H) and the protein expression of γ-H2AX (Figures 5I,J). To verify whether the upregulation of SIRT1 is involved in the protective effect of aspirin on cell senescence and DNA damage, we introduced a SIRT1 inhibitor, EX527. Aspirin reduced Aβ-increased SA-β-gal-positive cells, and this effect was blocked when EX527 was applied with aspirin (Figures 5K,L). Finally, the result of aspirin on DNA damage was also confirmed in SH-SY5Ycells (Figures 5M,N). These finding suggested that aspirin could suppress Aβ-induced cell senescence and DNA damage in neuronal cells partially depending on the upregulation of SIRT1.

## Discussion

Cell senescence plays an important role in the development of many age-related diseases, such as AD. Increased senescent cells are found in the brains of AD mouse models and patients with AD with high expression of Aβ and tau protein (Kritsilis et al., 2018). Some *in vitro* studies have also shown that Aβ could induce astrocytes, microglia, and endothelial cell senescence (Bhat et al., 2012; Caldeira et al., 2017; Singh Angom et al., 2019). For the neuronal cells, there are several lines of evidence demonstrated that the exposure of oligomeric Aβ accumulated p16 protein level but not induced the SA-β-gal activity in cultured mouse neuron (Wei et al., 2016), and the other two studies showed that Aβ could promote some cell senescence-associated phenotypes without the detection of DNA damage in cultured M17 neuronal cell line (Wang et al., 2021; Gu et al., 2022). In this study, we further demonstrated that Aβ is sufficient to accelerate cell senescence through upregulating p21 and PAI-1, increasing SA-β-gal-positive cells, and activating DNA damage response in multiple human neuronal cells including SK-N-SH cells, SH-SY5Y cells, and more relevant NSC-derived neuronal cells. Certainly, in addition to Aβ pathology, there are many other factors, including inflammation, tau pathology, APOE4 mutation, and mitochondrial dysfunction which may consequently trigger advanced cell senescence in the brain of patients with AD (Musi et al., 2018; Chapman et al., 2019; Guerrero et al., 2021).

The sirtuin family has beneficial effects on aging and AD. SIRT1 is widely reported to slow cell senescence in many different types of cells, including endothelial cells, fibroblast, and human mesenchymal stem cells (hMSCs) (Ota et al., 2007; Huang et al., 2008; Chen et al., 2014). Reduction of SIRT1 results in DNA damage response and cellular aging, whereas the research on human neuronal senescence and senescence-associated DNA damage has not been well documented in AD. Some chemicals have been shown to prevent Aβ-induced cell senescence through the modulation of SIRT1 (Wang et al., 2021; Gu et al., 2022) while whether SIRT1 can directly counteract Aβ effects on cellular senescence requires more investigations. Here, we found that Aβ treatment significantly declined SIRT1 protein expression in SK-N-SH cells in a time-dependent manner, and this result was consistently observed in SH-SY5Y cells and NSC-derived neuronal cells. Moreover, the reduction of SIRT1 by transfecting siRNA accelerated cell senescence, and the exogenous expression of SIRT1 reduced Aβ-induced senescence phenotypes and senescence-associated DNA damage response in both SK-N-SH and SH-SY5Y cells. These results revealed that modulating SIRT1 can regulate Aβ-induced senescence of human neuronal cells. Other than SIRT1, some other sirtuins play an essential factor in delaying cellular aging, such as SIRT3 (Diao et al., 2021) and SIRT6 (Nagai et al., 2015), and the underlying mechanism by which SIRT1 regulates Aβ-induced senescence requires further investigations.

Recent studies have reported that some clinically and preclinically used drugs, including metformin, rapamycin, resveratrol, and nicotinamide riboside (NR), have anti-aging effects. These chemicals can alleviate senescence phenotypes including the reduction of SA-β-gal, the decline of cell cycle genes, the suppression of DNA damage response, and the inhibition of inflammation through various signaling pathways. For example, metformin can reduce human cellular aging through upregulating GPx7 by Nrf2 signal pathway (Fang et al., 2018). Aspirin was observed to slower cognitive decline in patients with AD and to alleviate amyloid plaque pathology in an AD mouse model (Chandra et al., 2018; Weng et al., 2021). However, the effects on neuronal cell senescence are unknown in AD. Senolytics, a class of drugs that selectively clear senescent cells, are prevalently reported to reduce inflammation and SASP, improve tissue function, and prolong longevity (Xu M. et al., 2018; Zhang et al., 2019). These drugs can selectively induce apoptosis of senescent cells without influencing healthy cells. In our present study, we further demonstrated that aspirin reduced SA-β-gal-positive cells, p21 and PAI-1 expression, and DNA damage response partially through modulating SIRT1 in AD-associated human neuronal cells, suggesting that aspirin may be a potential drug for aging and age-related diseases. Of course, this needs to be further investigated using other methods such as gene editing.

In conclusion, this study revealed that Aβ accelerated cell senescence in multiple human neuronal cells, including SK-N-SH cells, SH-SY5Y cells, and human NSC-derived neuronal cells through downregulating SIRT1. Exogenous expression of SIRT1 rescued Aβ-induced cell senescence. In addition, aspirin reduced the levels of cell senescence markers at least partially through upregulating SIRT1, indicating that aspirin is possibly beneficial for aging-associated disorders.

## Data Availability Statement

The original contributions presented in the study are included in the article/Supplementary Material, further inquiries can be directed to the corresponding author/s.

## Ethics Statement

This study was approved by the Institutional Ethics Committee of the Shanghai Institute of Biochemistry and Cell Biology, Chinese Academy of Sciences.

## Author Contributions

GP and SH supervised the project and revised the manuscript. YL designed and conducted most experiments, analyzed data, organized figures and drafted manuscript. JL provided critical technique supports and data analysis. YH revised the manuscript. All authors approved the submitted manuscript.

## Conflict of Interest

The authors declare that the research was conducted in the absence of any commercial or financial relationships that could be construed as a potential conflict of interest.

## Publisher’s Note

All claims expressed in this article are solely those of the authors and do not necessarily represent those of their affiliated organizations, or those of the publisher, the editors and the reviewers. Any product that may be evaluated in this article, or claim that may be made by its manufacturer, is not guaranteed or endorsed by the publisher.
