# Supplementary material for: Alzheimer’s Amyloid-β Accelerates Human Neuronal Cell Senescence Which Could Be Rescued by Sirtuin-1 and Aspirin
Source: Front Cell Neurosci. 2022 Jun 17;16:906270. doi: 10.3389/fncel.2022.906270 (PMC9249263; doi:10.3389/fncel.2022.906270)
Supplement: Supplementary file 1 [file Data_Sheet_1.pdf]

Supplementary Material

Supplemental Figure1: Aβ promoted SA-β-gal positive cells in dose-dependently and the characterization of neuronal cells derived from neural stem cell (NSC).

(A-B) The representative images of SA-β-gal staining in SK-N-SH cells treated by Aβ at indicated dose (A). The images were captured by Olympus IX73. Scale bars, 50 µm. Quantification of relative number of SA-β-gal positive cells in (B).
(C) The representative images of characterization of neuronal cells derived from neural stem cell (NSC), stained at day14 of differentiation. Sox2 (marker of neural stem cell), Tuj1 (neuronal marker) The pictures were obtained by Leica TCS SP8 WLL. Scale bar, 50 µm.
The Data are presented as mean ± SEM, $n \geq 3$ independent experiments, ***$p< 0.001$ and ****$p< 0.0001$, analyzed by one-way ANOVA followed by Bonferroni test.

Supplemental Figure2: Aβ induced DNA damage response in NSC-derived neuronal cells and genomic DNA lesion in SK-N-SH and SH-SY5Y cells.

(A-B) Western blot analysis of γ-H2AX protein in NSC-derived neuronal cells treated by Aβ (5 µM) for 48 h. (A). Quantification of protein level of γ-H2AX (B).
(C) The representative images of agarose gel electrophoresis of the relative amplification of nuclear (12.2 kb and 13.5 kb) genomic DNA. 175 bp as an inner control.
(D) Cell were incubated with aspirin at indicated dose for 72 h. Then cell growth were detected using Luminescent cell viability Assay.
The Data are presented as mean ± SEM, $n \geq 3$ independent experiments, **$p < 0.01$, analyzed by one-way ANOVA followed by Bonferroni test.

Fig.S1.

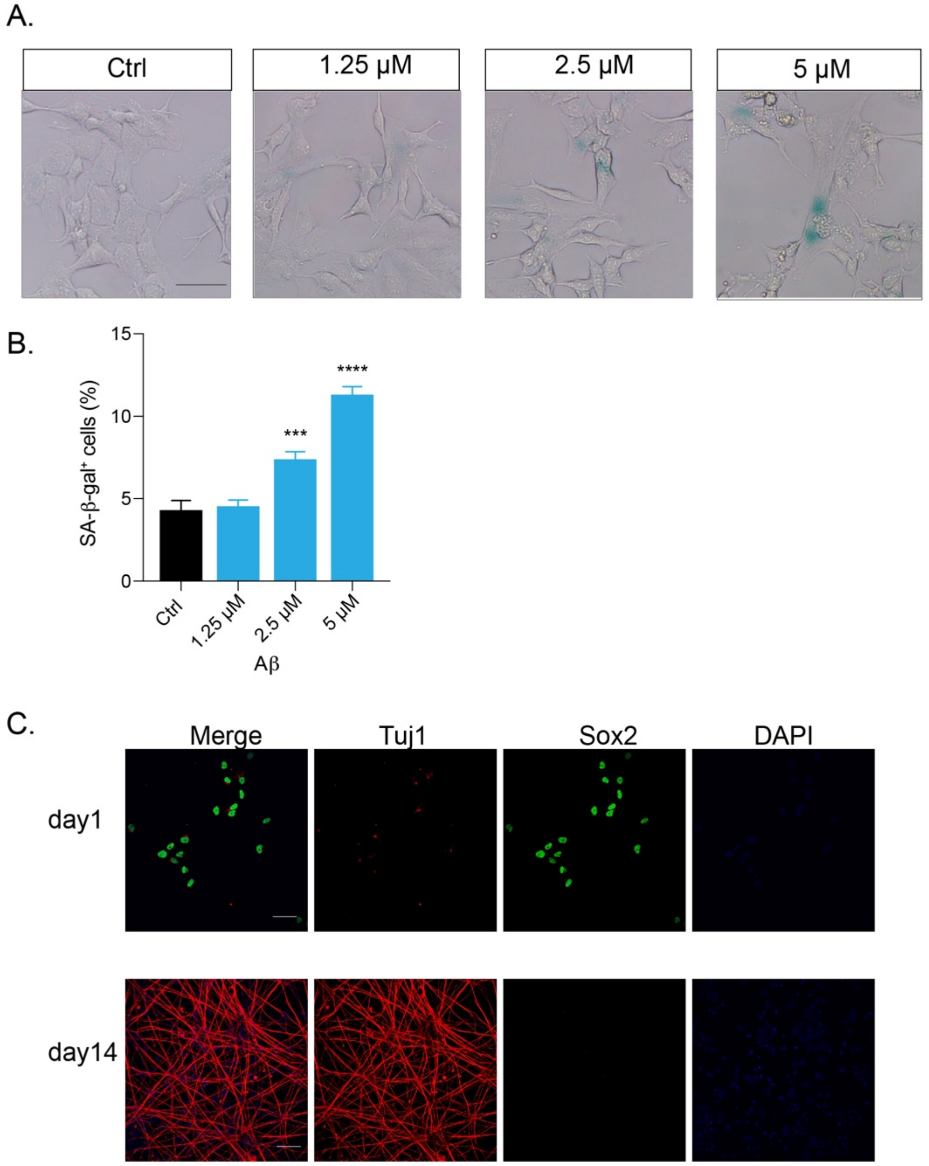
A.
Ctrl
1.25 μM
2.5 μM
5 μM
B.
SA-β-gal⁺ cells (%)
15
10
5
0
***
****
Ctrl
1.25 μM
2.5 μM
5 μM
Aβ
C.
Merge
Tuj1
Sox2
DAPI
day1
day14

Fig.S2.

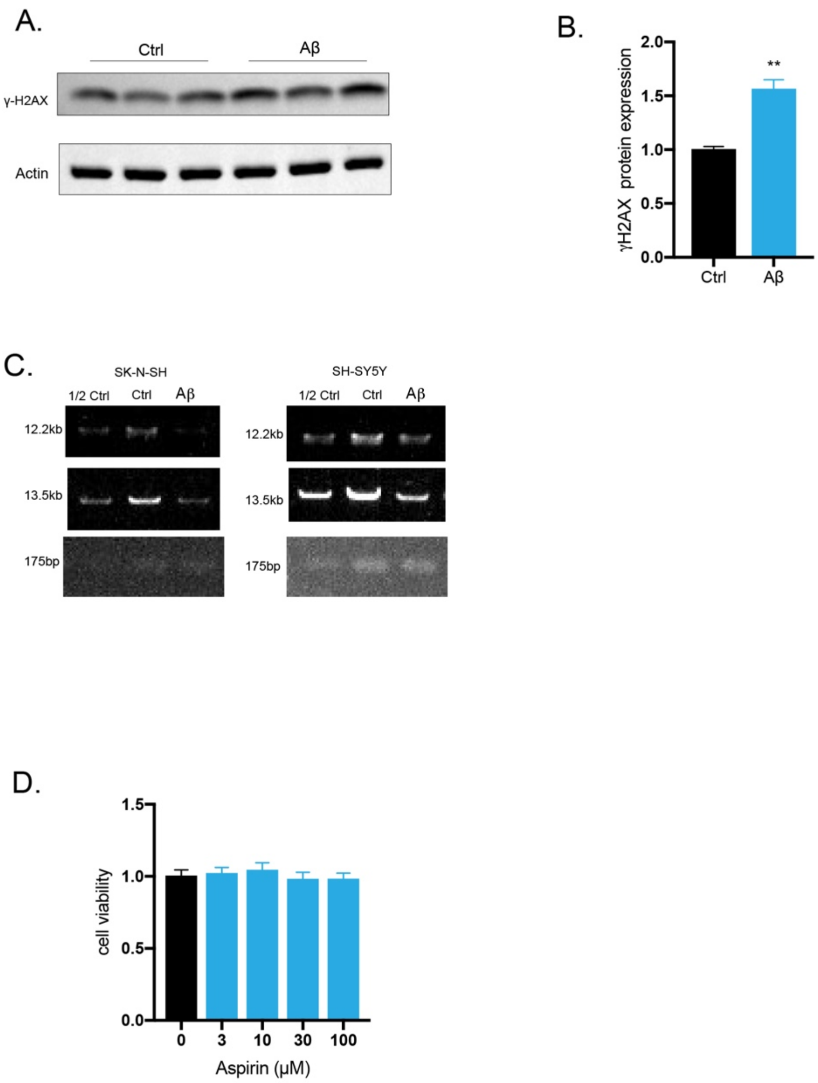
A.
Ctrl
Aβ
γ-H2AX
Actin
B.
γH2AX protein expression
2.0
1.5
1.0
0.5
0.0
**
Ctrl
Aβ
C.
SK-N-SH
1/2 Ctrl
Ctrl
Aβ
12.2kb
13.5kb
175bp
SH-SY5Y
1/2 Ctrl
Ctrl
Aβ
12.2kb
13.5kb
175bp
D.
cell viability
1.5
1.0
0.5
0.0
0
3
10
30
100
Aspirin (μM)
